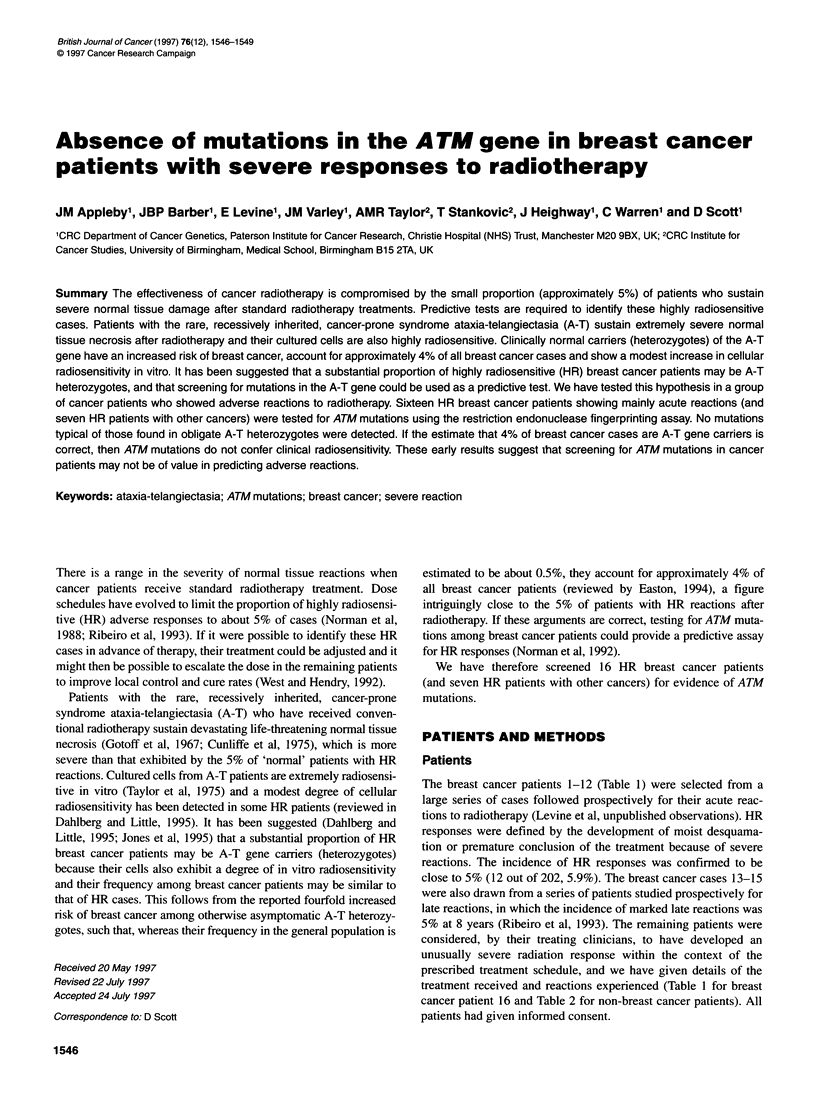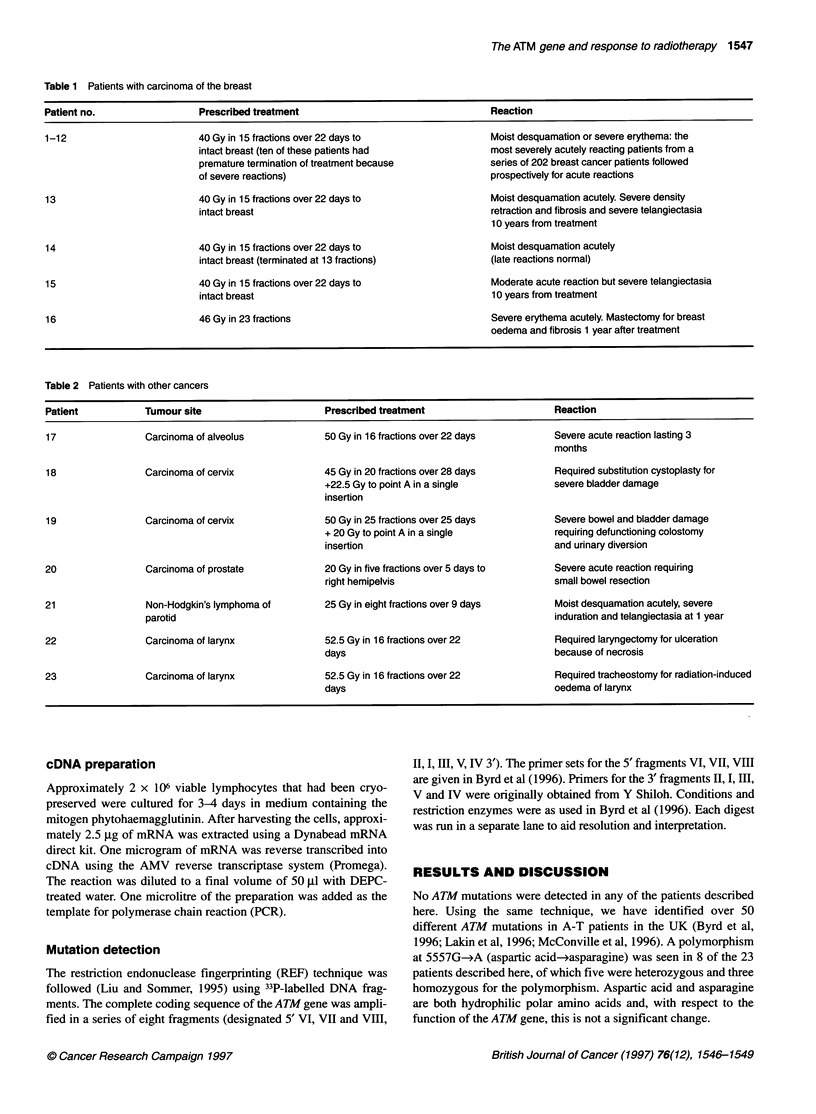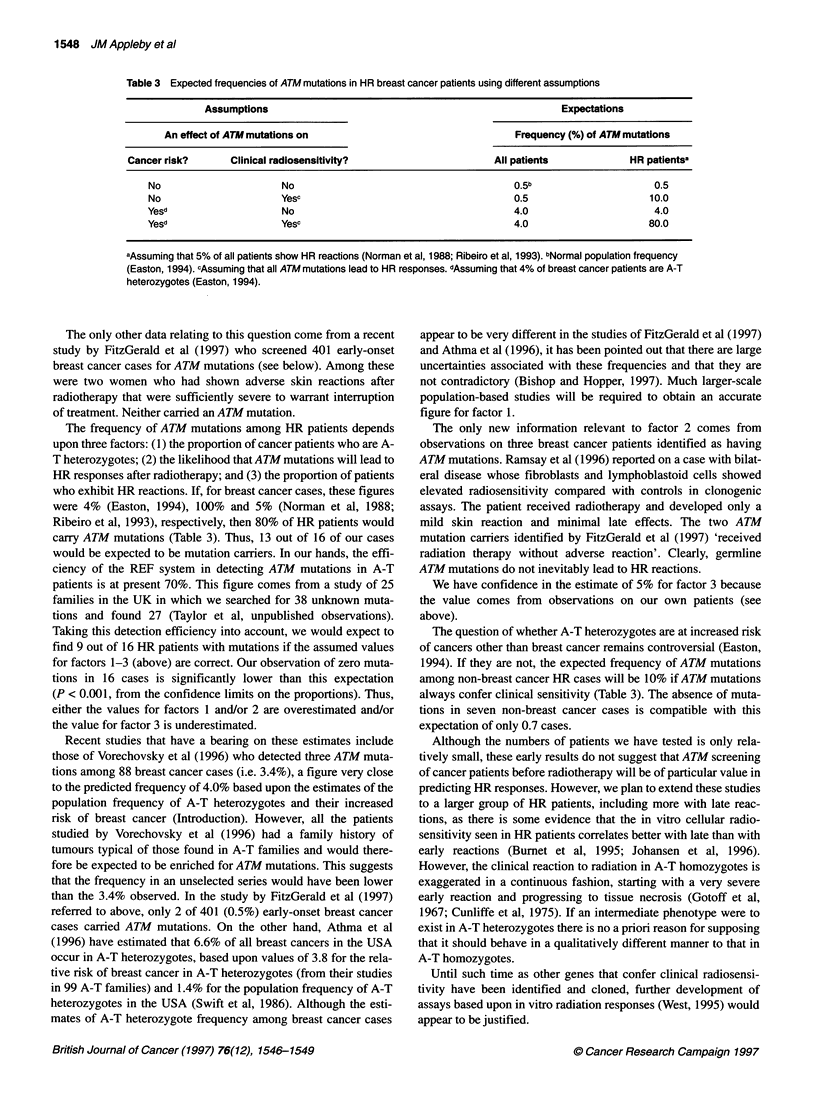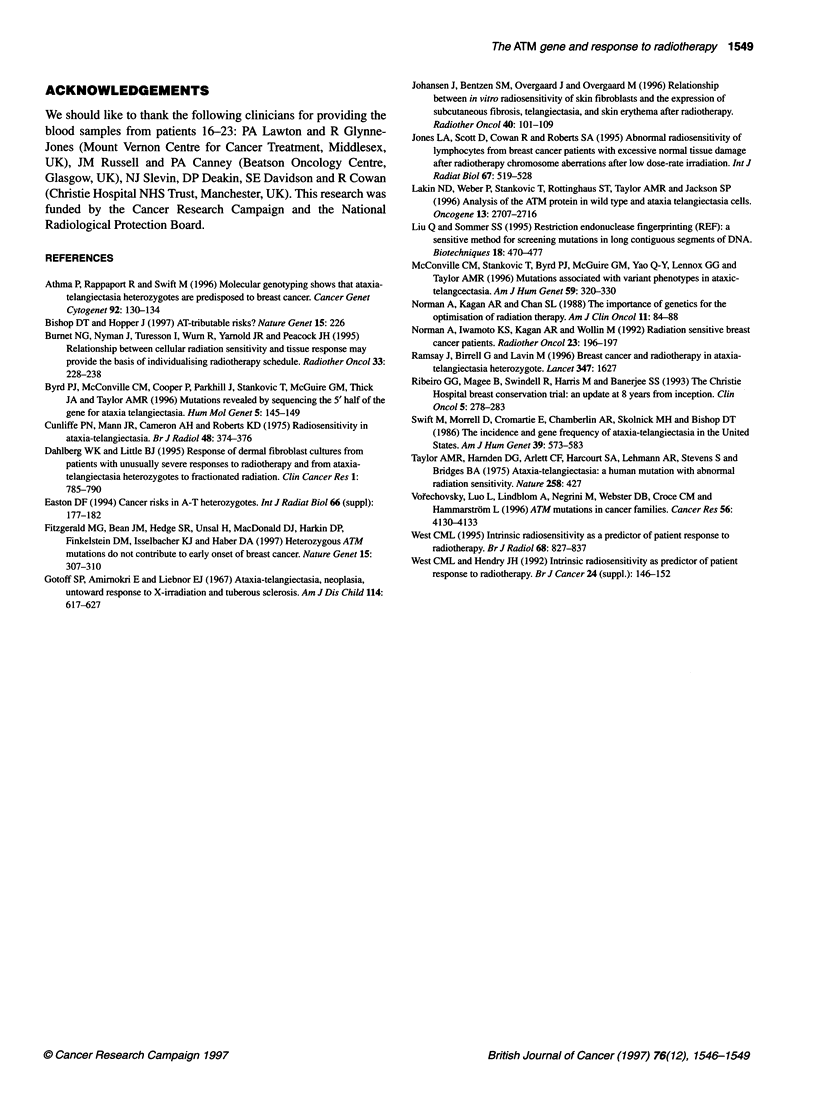# Absence of mutations in the ATM gene in breast cancer patients with severe responses to radiotherapy.

**DOI:** 10.1038/bjc.1997.593

**Published:** 1997

**Authors:** J. M. Appleby, J. B. Barber, E. Levine, J. M. Varley, A. M. Taylor, T. Stankovic, J. Heighway, C. Warren, D. Scott

**Affiliations:** CRC Department of Cancer Genetics, Paterson Institute for Cancer Research, Christie Hospital (NHS) Trust, Manchester, UK.

## Abstract

The effectiveness of cancer radiotherapy is compromised by the small proportion (approximately 5%) of patients who sustain severe normal tissue damage after standard radiotherapy treatments. Predictive tests are required to identify these highly radiosensitive cases. Patients with the rare, recessively inherited, cancer-prone syndrome ataxia-telangiectasia (A-T) sustain extremely severe normal tissue necrosis after radiotherapy and their cultured cells are also highly radiosensitive. Clinically normal carriers (heterozygotes) of the A-T gene have an increased risk of breast cancer, account for approximately 4% of all breast cancer cases and show a modest increase in cellular radiosensitivity in vitro. It has been suggested that a substantial proportion of highly radiosensitive (HR) breast cancer patients may be A-T heterozygotes, and that screening for mutations in the A-T gene could be used as a predictive test. We have tested this hypothesis in a group of cancer patients who showed adverse reactions to radiotherapy. Sixteen HR breast cancer patients showing mainly acute reactions (and seven HR patients with other cancers) were tested for ATM mutations using the restriction endonuclease fingerprinting assay. No mutations typical of those found in obligate A-T heterozygotes were detected. If the estimate that 4% of breast cancer cases are A-T gene carriers is correct, then ATM mutations do not confer clinical radiosensitivity. These early results suggest that screening for ATM mutations in cancer patients may not be of value in predicting adverse reactions.


British Journal of Cancer (1997) 76(12), 1546–1549
© 1997 Cancer Research Campaign

### Absence of mutations in the *ATM* gene in breast cancer patients with severe responses to radiotherapy

**JM Appleby[1], JBP Barber[1], E Levine[1], JM Varley[1], AMR Taylor[2], T Stankovic[2], J Heighway[1], C Warren[1] and D Scott[1]**

[1]CRC Department of Cancer Genetics, Paterson Institute for Cancer Research, Christie Hospital (NHS) Trust, Manchester M20 9BX, UK; [2]CRC Institute for Cancer Studies, University of Birmingham, Medical School, Birmingham B15 2TA, UK

**Summary** The effectiveness of cancer radiotherapy is compromised by the small proportion (approximately 5%) of patients who sustain severe normal tissue damage after standard radiotherapy treatments. Predictive tests are required to identify these highly radiosensitive cases. Patients with the rare, recessively inherited, cancer-prone syndrome ataxia-telangiectasia (A-T) sustain extremely severe normal tissue necrosis after radiotherapy and their cultured cells are also highly radiosensitive. Clinically normal carriers (heterozygotes) of the A-T gene have an increased risk of breast cancer, account for approximately 4% of all breast cancer cases and show a modest increase in cellular radiosensitivity in vitro. It has been suggested that a substantial proportion of highly radiosensitive (HR) breast cancer patients may be A-T heterozygotes, and that screening for mutations in the A-T gene could be used as a predictive test. We have tested this hypothesis in a group of cancer patients who showed adverse reactions to radiotherapy. Sixteen HR breast cancer patients showing mainly acute reactions (and seven HR patients with other cancers) were tested for *ATM* mutations using the restriction endonuclease fingerprinting assay. No mutations typical of those found in obligate A-T heterozygotes were detected. If the estimate that 4% of breast cancer cases are A-T gene carriers is correct, then *ATM* mutations do not confer clinical radiosensitivity. These early results suggest that screening for *ATM* mutations in cancer patients may not be of value in predicting adverse reactions.

**Keywords:** ataxia-telangiectasia; *ATM* mutations; breast cancer; severe reaction

There is a range in the severity of normal tissue reactions when cancer patients receive standard radiotherapy treatment. Dose schedules have evolved to limit the proportion of highly radiosensitive (HR) adverse responses to about 5% of cases (Norman et al, 1988; Ribeiro et al, 1993). If it were possible to identify these HR cases in advance of therapy, their treatment could be adjusted and it might then be possible to escalate the dose in the remaining patients to improve local control and cure rates (West and Hendry, 1992).

Patients with the rare, recessively inherited, cancer-prone syndrome ataxia-telangiectasia (A-T) who have received conventional radiotherapy sustain devastating life-threatening normal tissue necrosis (Gotoff et al, 1967; Cunliffe et al, 1975), which is more severe than that exhibited by the 5% of 'normal' patients with HR reactions. Cultured cells from A-T patients are extremely radiosensitive in vitro (Taylor et al, 1975) and a modest degree of cellular radiosensitivity has been detected in some HR patients (reviewed in Dahlberg and Little, 1995). It has been suggested (Dahlberg and Little, 1995; Jones et al, 1995) that a substantial proportion of HR breast cancer patients may be A-T gene carriers (heterozygotes) because their cells also exhibit a degree of in vitro radiosensitivity and their frequency among breast cancer patients may be similar to that of HR cases. This follows from the reported fourfold increased risk of breast cancer among otherwise asymptomatic A-T heterozygotes, such that, whereas their frequency in the general population is estimated to be about 0.5%, they account for approximately 4% of all breast cancer patients (reviewed by Easton, 1994), a figure intriguingly close to the 5% of patients with HR reactions after radiotherapy. If these arguments are correct, testing for *ATM* mutations among breast cancer patients could provide a predictive assay for HR responses (Norman et al, 1992).

We have therefore screened 16 HR breast cancer patients (and seven HR patients with other cancers) for evidence of *ATM* mutations.

*Received 20 May 1997*
*Revised 22 July 1997*
*Accepted 24 July 1997*

*Correspondence to:* D Scott

### PATIENTS AND METHODS

### Patients

The breast cancer patients 1–12 (Table 1) were selected from a large series of cases followed prospectively for their acute reactions to radiotherapy (Levine et al, unpublished observations). HR responses were defined by the development of moist desquamation or premature conclusion of the treatment because of severe reactions. The incidence of HR responses was confirmed to be close to 5% (12 out of 202, 5.9%). The breast cancer cases 13–15 were also drawn from a series of patients studied prospectively for late reactions, in which the incidence of marked late reactions was 5% at 8 years (Ribeiro et al, 1993). The remaining patients were considered, by their treating clinicians, to have developed an unusually severe radiation response within the context of the prescribed treatment schedule, and we have given details of the treatment received and reactions experienced (Table 1 for breast cancer patient 16 and Table 2 for non-breast cancer patients). All patients had given informed consent.

**Table 1** Patients with carcinoma of the breast

| Patient no. | Prescribed treatment | Reaction |
|---|---|---|
| 1–12 | 40 Gy in 15 fractions over 22 days to intact breast (ten of these patients had premature termination of treatment because of severe reactions) | Moist desquamation or severe erythema: the most severely acutely reacting patients from a series of 202 breast cancer patients followed prospectively for acute reactions |
| 13 | 40 Gy in 15 fractions over 22 days to intact breast | Moist desquamation acutely. Severe density retraction and fibrosis and severe telangiectasia 10 years from treatment |
| 14 | 40 Gy in 15 fractions over 22 days to intact breast (terminated at 13 fractions) | Moist desquamation acutely (late reactions normal) |
| 15 | 40 Gy in 15 fractions over 22 days to intact breast | Moderate acute reaction but severe telangiectasia 10 years from treatment |
| 16 | 46 Gy in 23 fractions | Severe erythema acutely. Mastectomy for breast oedema and fibrosis 1 year after treatment |

**Table 2** Patients with other cancers

| Patient | Tumour site | Prescribed treatment | Reaction |
|---|---|---|---|
| 17 | Carcinoma of alveolus | 50 Gy in 16 fractions over 22 days | Severe acute reaction lasting 3 months |
| 18 | Carcinoma of cervix | 45 Gy in 20 fractions over 28 days +22.5 Gy to point A in a single insertion | Required substitution cystoplasty for severe bladder damage |
| 19 | Carcinoma of cervix | 50 Gy in 25 fractions over 25 days + 20 Gy to point A in a single insertion | Severe bowel and bladder damage requiring defunctioning colostomy and urinary diversion |
| 20 | Carcinoma of prostate | 20 Gy in five fractions over 5 days to right hemipelvis | Severe acute reaction requiring small bowel resection |
| 21 | Non-Hodgkin's lymphoma of parotid | 25 Gy in eight fractions over 9 days | Moist desquamation acutely, severe induration and telangiectasia at 1 year |
| 22 | Carcinoma of larynx | 52.5 Gy in 16 fractions over 22 days | Required laryngectomy for ulceration because of necrosis |
| 23 | Carcinoma of larynx | 52.5 Gy in 16 fractions over 22 days | Required tracheostomy for radiation-induced oedema of larynx |

### cDNA preparation

Approximately $2 \times 10^6$ viable lymphocytes that had been cryopreserved were cultured for 3–4 days in medium containing the mitogen phytohaemagglutinin. After harvesting the cells, approximately 2.5 μg of mRNA was extracted using a Dynabead mRNA direct kit. One microgram of mRNA was reverse transcribed into cDNA using the AMV reverse transcriptase system (Promega). The reaction was diluted to a final volume of 50 μl with DEPC-treated water. One microlitre of the preparation was added as the template for polymerase chain reaction (PCR).

### Mutation detection

The restriction endonuclease fingerprinting (REF) technique was followed (Liu and Sommer, 1995) using $^{33}$P-labelled DNA fragments. The complete coding sequence of the *ATM* gene was amplified in a series of eight fragments (designated 5′ VI, VII and VIII, II, I, III, V, IV 3′). The primer sets for the 5′ fragments VI, VII, VIII are given in Byrd et al (1996). Primers for the 3′ fragments II, I, III, V and IV were originally obtained from Y Shiloh. Conditions and restriction enzymes were as used in Byrd et al (1996). Each digest was run in a separate lane to aid resolution and interpretation.

### RESULTS AND DISCUSSION

No *ATM* mutations were detected in any of the patients described here. Using the same technique, we have identified over 50 different *ATM* mutations in A-T patients in the UK (Byrd et al, 1996; Lakin et al, 1996; McConville et al, 1996). A polymorphism at 5557G→A (aspartic acid→asparagine) was seen in 8 of the 23 patients described here, of which five were heterozygous and three homozygous for the polymorphism. Aspartic acid and asparagine are both hydrophilic polar amino acids and, with respect to the function of the *ATM* gene, this is not a significant change.

© Cancer Research Campaign 1997 

**Table 3** Expected frequencies of *ATM* mutations in HR breast cancer patients using different assumptions

| Assumptions | | Expectations | |
|---|---|---|---|
| An effect of *ATM* mutations on | | Frequency (%) of *ATM* mutations | |
| Cancer risk? | Clinical radiosensitivity? | All patients | HR patients[a] |
| No | No | 0.5[b] | 0.5 |
| No | Yes[c] | 0.5 | 10.0 |
| Yes[d] | No | 4.0 | 4.0 |
| Yes[d] | Yes[c] | 4.0 | 80.0 |

[a]Assuming that 5% of all patients show HR reactions (Norman et al, 1988; Ribeiro et al, 1993). [b]Normal population frequency (Easton, 1994). [c]Assuming that all *ATM* mutations lead to HR responses. [d]Assuming that 4% of breast cancer patients are A-T heterozygotes (Easton, 1994).

The only other data relating to this question come from a recent study by FitzGerald et al (1997) who screened 401 early-onset breast cancer cases for *ATM* mutations (see below). Among these were two women who had shown adverse skin reactions after radiotherapy that were sufficiently severe to warrant interruption of treatment. Neither carried an *ATM* mutation.

The frequency of *ATM* mutations among HR patients depends upon three factors: (1) the proportion of cancer patients who are A-T heterozygotes; (2) the likelihood that *ATM* mutations will lead to HR responses after radiotherapy; and (3) the proportion of patients who exhibit HR reactions. If, for breast cancer cases, these figures were 4% (Easton, 1994), 100% and 5% (Norman et al, 1988; Ribeiro et al, 1993), respectively, then 80% of HR patients would carry *ATM* mutations (Table 3). Thus, 13 out of 16 of our cases would be expected to be mutation carriers. In our hands, the efficiency of the REF system in detecting *ATM* mutations in A-T patients is at present 70%. This figure comes from a study of 25 families in the UK in which we searched for 38 unknown mutations and found 27 (Taylor et al, unpublished observations). Taking this detection efficiency into account, we would expect to find 9 out of 16 HR patients with mutations if the assumed values for factors 1–3 (above) are correct. Our observation of zero mutations in 16 cases is significantly lower than this expectation ($P < 0.001$, from the confidence limits on the proportions). Thus, either the values for factors 1 and/or 2 are overestimated and/or the value for factor 3 is underestimated.

Recent studies that have a bearing on these estimates include those of Vorechovsky et al (1996) who detected three *ATM* mutations among 88 breast cancer cases (i.e. 3.4%), a figure very close to the predicted frequency of 4.0% based upon the estimates of the population frequency of A-T heterozygotes and their increased risk of breast cancer (Introduction). However, all the patients studied by Vorechovsky et al (1996) had a family history of tumours typical of those found in A-T families and would therefore be expected to be enriched for *ATM* mutations. This suggests that the frequency in an unselected series would have been lower than the 3.4% observed. In the study by FitzGerald et al (1997) referred to above, only 2 of 401 (0.5%) early-onset breast cancer cases carried *ATM* mutations. On the other hand, Athma et al (1996) have estimated that 6.6% of all breast cancers in the USA occur in A-T heterozygotes, based upon values of 3.8 for the relative risk of breast cancer in A-T heterozygotes (from their studies in 99 A-T families) and 1.4% for the population frequency of A-T heterozygotes in the USA (Swift et al, 1986). Although the estimates of A-T heterozygote frequency among breast cancer cases appear to be very different in the studies of FitzGerald et al (1997) and Athma et al (1996), it has been pointed out that there are large uncertainties associated with these frequencies and that they are not contradictory (Bishop and Hopper, 1997). Much larger-scale population-based studies will be required to obtain an accurate figure for factor 1.

The only new information relevant to factor 2 comes from observations on three breast cancer patients identified as having *ATM* mutations. Ramsay et al (1996) reported on a case with bilateral disease whose fibroblasts and lymphoblastoid cells showed elevated radiosensitivity compared with controls in clonogenic assays. The patient received radiotherapy and developed only a mild skin reaction and minimal late effects. The two *ATM* mutation carriers identified by FitzGerald et al (1997) 'received radiation therapy without adverse reaction'. Clearly, germline *ATM* mutations do not inevitably lead to HR reactions.

We have confidence in the estimate of 5% for factor 3 because the value comes from observations on our own patients (see above).

The question of whether A-T heterozygotes are at increased risk of cancers other than breast cancer remains controversial (Easton, 1994). If they are not, the expected frequency of *ATM* mutations among non-breast cancer HR cases will be 10% if *ATM* mutations always confer clinical sensitivity (Table 3). The absence of mutations in seven non-breast cancer cases is compatible with this expectation of only 0.7 cases.

Although the numbers of patients we have tested is only relatively small, these early results do not suggest that *ATM* screening of cancer patients before radiotherapy will be of particular value in predicting HR responses. However, we plan to extend these studies to a larger group of HR patients, including more with late reactions, as there is some evidence that the in vitro cellular radiosensitivity seen in HR patients correlates better with late than with early reactions (Burnet et al, 1995; Johansen et al, 1996). However, the clinical reaction to radiation in A-T homozygotes is exaggerated in a continuous fashion, starting with a very severe early reaction and progressing to tissue necrosis (Gotoff et al, 1967; Cunliffe et al, 1975). If an intermediate phenotype were to exist in A-T heterozygotes there is no a priori reason for supposing that it should behave in a qualitatively different manner to that in A-T homozygotes.

Until such time as other genes that confer clinical radiosensitivity have been identified and cloned, further development of assays based upon in vitro radiation responses (West, 1995) would appear to be justified.

© Cancer Research Campaign 1997

### ACKNOWLEDGEMENTS

We should like to thank the following clinicians for providing the blood samples from patients 16–23: PA Lawton and R Glynne-Jones (Mount Vernon Centre for Cancer Treatment, Middlesex, UK), JM Russell and PA Canney (Beatson Oncology Centre, Glasgow, UK), NJ Slevin, DP Deakin, SE Davidson and R Cowan (Christie Hospital NHS Trust, Manchester, UK). This research was funded by the Cancer Research Campaign and the National Radiological Protection Board.

### REFERENCES

Athma P, Rappaport R and Swift M (1996) Molecular genotyping shows that ataxia-telangiectasia heterozygotes are predisposed to breast cancer. *Cancer Genet Cytogenet* **92**: 130–134

Bishop DT and Hopper J (1997) AT-tributable risks? *Nature Genet* **15**: 226

Burnet NG, Nyman J, Turesson I, Wurn R, Yarnold JR and Peacock JH (1995) Relationship between cellular radiation sensitivity and tissue response may provide the basis of individualising radiotherapy schedule. *Radiother Oncol* **33**: 228–238

Byrd PJ, McConville CM, Cooper P, Parkhill J, Stankovic T, McGuire GM, Thick JA and Taylor AMR (1996) Mutations revealed by sequencing the 5′ half of the gene for ataxia telangiectasia. *Hum Mol Genet* **5**: 145–149

Cunliffe PN, Mann JR, Cameron AH and Roberts KD (1975) Radiosensitivity in ataxia-telangiectasia. *Br J Radiol* **48**: 374–376

Dahlberg WK and Little BJ (1995) Response of dermal fibroblast cultures from patients with unusually severe responses to radiotherapy and from ataxia-telangiectasia heterozygotes to fractionated radiation. *Clin Cancer Res* **1**: 785–790

Easton DF (1994) Cancer risks in A-T heterozygotes. *Int J Radiat Biol* **66** (suppl): 177–182

Fitzgerald MG, Bean JM, Hedge SR, Unsal H, MacDonald DJ, Harkin DP, Finkelstein DM, Isselbacher KJ and Haber DA (1997) Heterozygous *ATM* mutations do not contribute to early onset of breast cancer. *Nature Genet* **15**: 307–310

Gotoff SP, Amirnokri E and Liebnor EJ (1967) Ataxia-telangiectasia, neoplasia, untoward response to X-irradiation and tuberous sclerosis. *Am J Dis Child* **114**: 617–627

Johansen J, Bentzen SM, Overgaard J and Overgaard M (1996) Relationship between *in vitro* radiosensitivity of skin fibroblasts and the expression of subcutaneous fibrosis, telangiectasia, and skin erythema after radiotherapy. *Radiother Oncol* **40**: 101–109

Jones LA, Scott D, Cowan R and Roberts SA (1995) Abnormal radiosensitivity of lymphocytes from breast cancer patients with excessive normal tissue damage after radiotherapy chromosome aberrations after low dose-rate irradiation. *Int J Radiat Biol* **67**: 519–528

Lakin ND, Weber P, Stankovic T, Rottinghaus ST, Taylor AMR and Jackson SP (1996) Analysis of the ATM protein in wild type and ataxia telangiectasia cells. *Oncogene* **13**: 2707–2716

Liu Q and Sommer SS (1995) Restriction endonuclease fingerprinting (REF): a sensitive method for screening mutations in long contiguous segments of DNA. *Biotechniques* **18**: 470–477

McConville CM, Stankovic T, Byrd PJ, McGuire GM, Yao Q-Y, Lennox GG and Taylor AMR (1996) Mutations associated with variant phenotypes in ataxic-telangcectasia. *Am J Hum Genet* **59**: 320–330

Norman A, Kagan AR and Chan SL (1988) The importance of genetics for the optimisation of radiation therapy. *Am J Clin Oncol* **11**: 84–88

Norman A, Iwamoto KS, Kagan AR and Wollin M (1992) Radiation sensitive breast cancer patients. *Radiother Oncol* **23**: 196–197

Ramsay J, Birrell G and Lavin M (1996) Breast cancer and radiotherapy in ataxia-telangiectasia heterozygote. *Lancet* **347**: 1627

Ribeiro GG, Magee B, Swindell R, Harris M and Banerjee SS (1993) The Christie Hospital breast conservation trial: an update at 8 years from inception. *Clin Oncol* **5**: 278–283

Swift M, Morrell D, Cromartie E, Chamberlin AR, Skolnick MH and Bishop DT (1986) The incidence and gene frequency of ataxia-telangiectasia in the United States. *Am J Hum Genet* **39**: 573–583

Taylor AMR, Harnden DG, Arlett CF, Harcourt SA, Lehmann AR, Stevens S and Bridges BA (1975) Ataxia-telangiectasia: a human mutation with abnormal radiation sensitivity. *Nature* **258**: 427

Vořechovsky, Luo L, Lindblom A, Negrini M, Webster DB, Croce CM and Hammarström L (1996) *ATM* mutations in cancer families. *Cancer Res* **56**: 4130–4133

West CML (1995) Intrinsic radiosensitivity as a predictor of patient response to radiotherapy. *Br J Radiol* **68**: 827–837

West CML and Hendry JH (1992) Intrinsic radiosensitivity as predictor of patient response to radiotherapy. *Br J Cancer* **24** (suppl.): 146–152

© Cancer Research Campaign 1997